# Bioinformatic Analysis Reveals Hub Immune-Related Genes of Diabetic Foot Ulcers

**DOI:** 10.3389/fsurg.2022.878965

**Published:** 2022-04-05

**Authors:** Yanchao Rong, Hao Yang, Hailin Xu, Shuting Li, Peng Wang, Zhiyong Wang, Yi Zhang, Wenkai Zhu, Bing Tang, Jiayuan Zhu, Zhicheng Hu

**Affiliations:** ^1^Department of Burn Surgery, The First Affiliated Hospital of Sun Yat-sen University, Guangzhou, China; ^2^Department of Plastic Surgery, The First Affiliated Hospital of Sun Yat-sen University, Guangzhou, China; ^3^Department of Burn and Plastic Surgery, Affiliated Hospital of Nantong University, Nantong, China; ^4^Department of Obstetrics and Gynecology, School of Medicine, Stanford University, Stanford, CA, United States

**Keywords:** diabetic foot ulcers, bioinformatics analysis, immune, wound repair, wound healing

## Abstract

Diabetic foot ulcer (DFU) is a complex and devastating complication of diabetes mellitus that are usually stagnant in the inflammatory phase. However, oral wound healing, which is characterized by a rapid and scarless healing process, is regarded an ideal model of wound healing. Thus, we performed a comprehensive bioinformatics analysis of the previously published data regarding oral ulcers and DFUs and found that compared to oral wound healing, the activated pathways of DFUs were enriched in cellular metabolism-related pathways but lacked the activation of inflammatory and immune-related pathways. We also found that CXCL11, DDX60, IFI44, and IFI44L were remarkable nodes since they had the most connections with other members of the module. Meanwhile, CXCL10, IRF7, and DDX58 together formed a closed-loop relationship and occupied central positions in the entire network. The real-time polymerase chain reaction and western blot was applied to validate the gene expression of the hub immune-related genes in the DFU tissues, it was found that CXCL11, IFI44, IFI44L, CXCL10 and IRF7 have a significant difference compared with normal wound tissues. Our research reveals some novel potential immune-related biomarkers and provides new insights into the molecular basis of this debilitating disease.

## Introduction

Diabetic foot ulcer (DFU) is a complex and devastating complication of diabetes mellitus. Diabetic patients have a 15–25% chance of developing DFUs in their lifetime and a 50–70% recurrence rate in 5 years (1, 2). The refractory wound of DFUs often leads to high rates of hospitalization, amputation, and mortality. At least 25% of DFUs do not heal and 28% may result in amputation (3). In 2016, 130,000 hospital discharges for lower extremity amputation due to DFUs were reported among US adults. Meanwhile, the health care costs of DFUs pose a considerable financial burden worldwide. In England, the health care cost of DFU and the consequent amputation was estimated to be between £837 million to £962 million in 2014, which is more than the combined health care cost of breast, prostate, and lung cancers (4). In the United States, diabetes and its complications generated a total health care cost of $237 billion in 2017 (2), of which 33% was related to the treatment of DFUs (5). Unfortunately, the health care cost of DFUs is expected to increase over the coming decades. Therefore, it is crucial to elucidate the molecular mechanisms that influence DFU progression in order to help prevent and treat these debilitating wounds.

Wound healing is a complicated process that can be divided into four integrated and overlapped phases: hemostasis, inflammation, tissue formation, and remodeling (6). Compared to the smooth and programmed healing process of acute wounds, diabetic wounds are usually stagnant in the inflammatory phase (7). After injury, neutrophils, the first inflammatory cells migrating to the wound area, clear dead cells and infectious microorganisms (8). The deregulation of neutrophil extracellular traps (NETs) activation and release (NETosis) process have been observed in the neutrophils isolated from patients with DFUs (9, 10). Afterward, monocytes appear in the wound area and are converted into macrophages (11). However, diabetes impairs the recruitment of monocytes, reduces phagocytosis, and prevents pro-inflammatory macrophages from turning into its anti-inflammatory phenotype (12). Thus, the prolonged inflammatory phase may lead to the chronic nature of DFUs wound healing. The concentration of pro-inflammatory cytokines, such as tumor necrosis factor α (TNF-α) and interleukin-1b (IL-1b), is increased and granulation and re-epithelialization are impaired in diabetic wounds (13). Therefore, maintaining a balanced inflammatory response is essential for the wound healing process.

Oral and embryonic wound healing, characterized by a rapid and scarless healing process, is regarded an ideal model of wound healing (6, 14, 15). The general principles and cellular and molecular mechanisms of skin wound healing also apply to the oral wound healing process. Hemostasis is followed by the early-stage of inflammation. Within hours of injury, the bacteria and necrotic tissues in the wound area is cleared by inflammatory cells (mostly neutrophils and monocytes) through accumulation, phagocytosis, and secretion of enzymes and toxic oxygen products (16). Within 3 days, the inflammation proceeds into its late stage. Macrophages release inflammatory cytokines and growth factors to remove apoptotic neutrophils, augment the inflammatory response, and initiate the granulation tissue formation (8, 17). Epithelialization is initiated within hours of an injury. Epithelial cells from the basal layer proliferate and migrate through the fibrin clot and eventually ensure that the wound is closed (18). Furthermore, in contrast to DFU wound, a high level of inflammatory response, followed by resolution of the inflammation (which ensures that recruited inflammatory cells are cleared from the site of injury), rapidly occurs to prevent chronic inflammation (19).

There are significant inflammatory responses in both oral and acute skin trauma, but oral mucosa has a higher inflammatory response than skin. Long-term chronic wounds have suboptimal levels of inflammation that are insufficient to promote healing. Therefore, stimulating an adequate inflammatory response can lead to better healing outcomes. In this study, we explored new immune-related gene biomarkers to improve immune status and accelerate chronic diabetic wound healing. Therefore, we performed a comprehensive bioinformatics analysis of the previously published data regarding oral ulcers and DFUs to explore the potential mechanisms of related biomarkers. Our results reveal novel potential immune-related biomarkers and provide new insights into the molecular basis of this debilitating disease.

## Materials and Methods

### Dataset Selection

The gene expression profiles GSE37265 and GSE80178 were retrieved from the gene expression omnibus (GEO) database (https://www.ncbi.nlm.nih.gov/geo/). The GSE37265 expression profile comprises 14 oral ulcers and five normal tissues and was analyzed by the Affymetrix Human Genome U133 Plus 2.0 Array. The GSE80178 expression profile comprises six DFUs and three normal skin tissues and was analyzed by the Affymetrix Human Gene 2.0 ST Array.

### Identification of Differentially Expressed Genes

The limma (linear models for microarray data) package in R (20) was used to identify DEGs, and *P*-values were adjusted by the Benjamini and Hochberg method. Transcripts with fold expression >2.0 and adjusted *P* < 0.05 between the two groups were considered significantly different. The results are presented as volcano plots and heat maps.

### Functional Enrichment Analysis

Functional enrichment analysis of DEGs was performed by the database for annotation, visualization, and integrated discovery (DAVID) (21). The DAVID was used to perform the Kyoto encyclopedia of genes and genomes (KEGG) pathway enrichment analysis. *P* < 0.05 and false discovery rates <0.25 were considered statistically significant.

### Immune Score and Immune Cell Infiltration Analyses

The ESTIMATE algorithm was applied to calculate the scores for the level of immune cells in every sample (22). Immune cell infiltration was evaluated by the CIBERSORT algorithm (23). CIBERSORT is an analysis tool that represents the cell composition of complex tissues through analyzing gene expression data according to pre-processed gene expression profiles.

### Protein-Protein Interaction Network Analysis

The PPI network was retrieved from the STRING database and reconstructed *via* the Cytoscape software (24, 25). The degree of connectivity of each node of the network was calculated. Afterward, we used molecular complex detection (MCODE) to find clusters according to topology locating densely connected regions.

### Weighted Gene Co-expression Network Analysis

GEO expression file was examined by WGCNA using the WGCNA package in R (26). WGCNA was used to explore the relationship between the immune score and expression modules. Furthermore, the correlation between genes in corresponding modules and gene expression profiles was defined as module membership (MM)., Each important gene's MM and gene significance (GS) were determined once the modules of interest were selected. Hub genes in each module were screened by establishing the thresholds of MM > 0.8 and GS > 0.1.

### RNA Extraction and Real-Time Polymerase Chain Reaction

DFU tissues and normal wound tissues were harvested during surgery and immediately frozen on dry ice and stored at– 80°C (*n* = 3 at each group). Total RNA extracted from rapidly frozen DRG tissues was isolated using Trizol reagent (Invitrogen). To analyze the expression level of CXCL11, DDX60, IFI44, IFI44L, CXCL10, IRF7, and DDX58, real-time polymerase chain reaction (PCR) was performed by using TaKaRa SYBR Premix Ex Taq II (Perfect Real Time). Specific primers for each gene were listed in Figure 7A.

### Protein Extraction and Western Blot

Total protein was extracted using a tissue protein extraction reagent (Beyotime Institute of Biotechnology, China) according to the manufacturer's instructions. Proteins were separated on the 8 or 7% SDS-polyacrylamide gel, then transferred to a PVDF membrane (Millipore, Bedford, MA). Subsequently, the membrane was blocked for 1 h in 5% skim milk at room temperature and probed with primary antibodies overnight in 0.5% skim milk at 4°C, followed by the incubation of horseradish-peroxidase-conjugated secondary antibodies for 1 h in 5% blocking buffer at room temperature. Protein bands were detected using a ECL chemiluminescence kit (Thermo Scientific, MA) with Image Quant LAS4000mini (GE Healthcare Life Sciences, UT) system. Primary and secondary antibodies are shown in Supplementary Table 1.

### Statistical Analyses

Statistical analyses were performed using Graph Pad Prism 7. Paired Samples test was used to compare the data between pairs of groups. *P* < 0.05 was considered statistically significant.

## Results

### Evaluation of Immune Status in GSE37265

**Figure 1:**
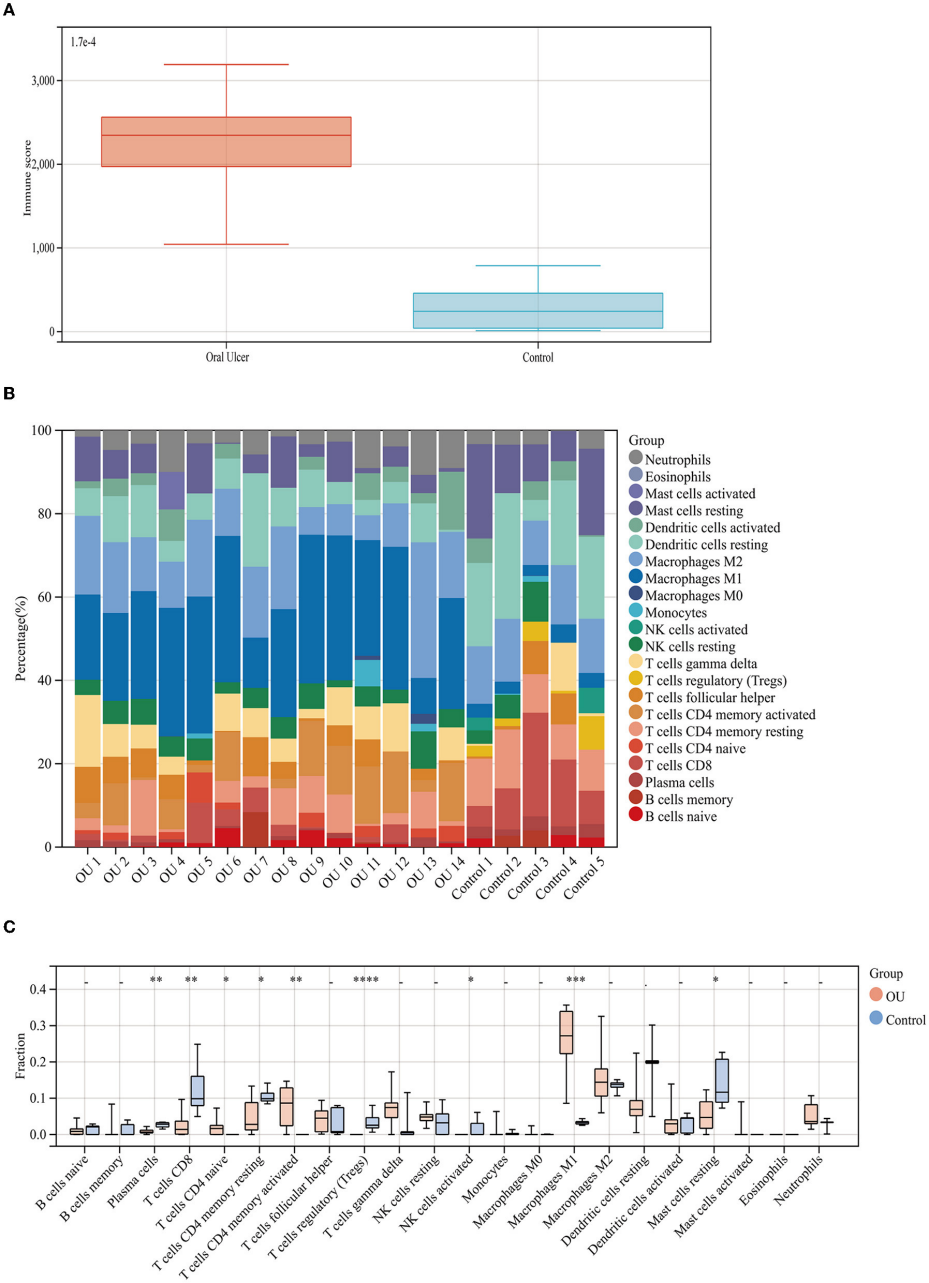
Immune score and immune cell infiltration analyses. **(A)** Distribution of immune scores of oral mucosal ulcers and normal oral mucosal tissues. **(B)** The fraction of 22 subsets of immune cells from oral mucosal ulcers and normal oral mucosal tissues. **(C)** Box-plot showing the difference in immune infiltration between oral mucosal ulcers and normal oral mucosal tissues. * < 0.05, ** < 0.01, *** < 0.005, **** < 0.001.

At first, we downloaded the GSE37265 dataset from the GEO database, which contains transcriptomic data of oral mucosal ulcers and normal oral mucosal tissues. The immune score of the two groups was calculated by the ESTIMATE algorithm and it was found that the immune score of the oral ulcers group was significantly higher than that of the control group (*P* < 0.05) (Figure 1A). Distribution of immune scores were shown in Supplementary Table 2. Afterward, we used the CIBERSORT algorithm to define 22 immune cell subpopulations and analyze the data from the oral ulcer and control groups. As shown in Figure 1B, the fraction of immune cells varied significantly among the samples and between the groups. In oral ulcers, M1 macrophages, M2 macrophages, and CD4 memory-activated T cells represented the top three highest infiltrating fractions. Inversely, T regulatory cells (Tregs), activated natural killer (NK) cells, and eosinophils were presented in lower quantities. Meanwhile, oral mucosal ulcers contained a higher fraction of CD4 naive T cells, CD4 memory-activated T cells, and M1 macrophages (*P* < 0.05) compared with normal oral mucosal tissues. However, oral mucosal ulcers contained a lower fraction of plasma cells, CD8 T cells, CD4 memory resting T cells, Tregs, activated NK cells, and resting Mast cells (*P* < 0.05) (Figure 1C).

### Identification and Functional Annotations of DEGs in GSE37265

**Figure 2:**
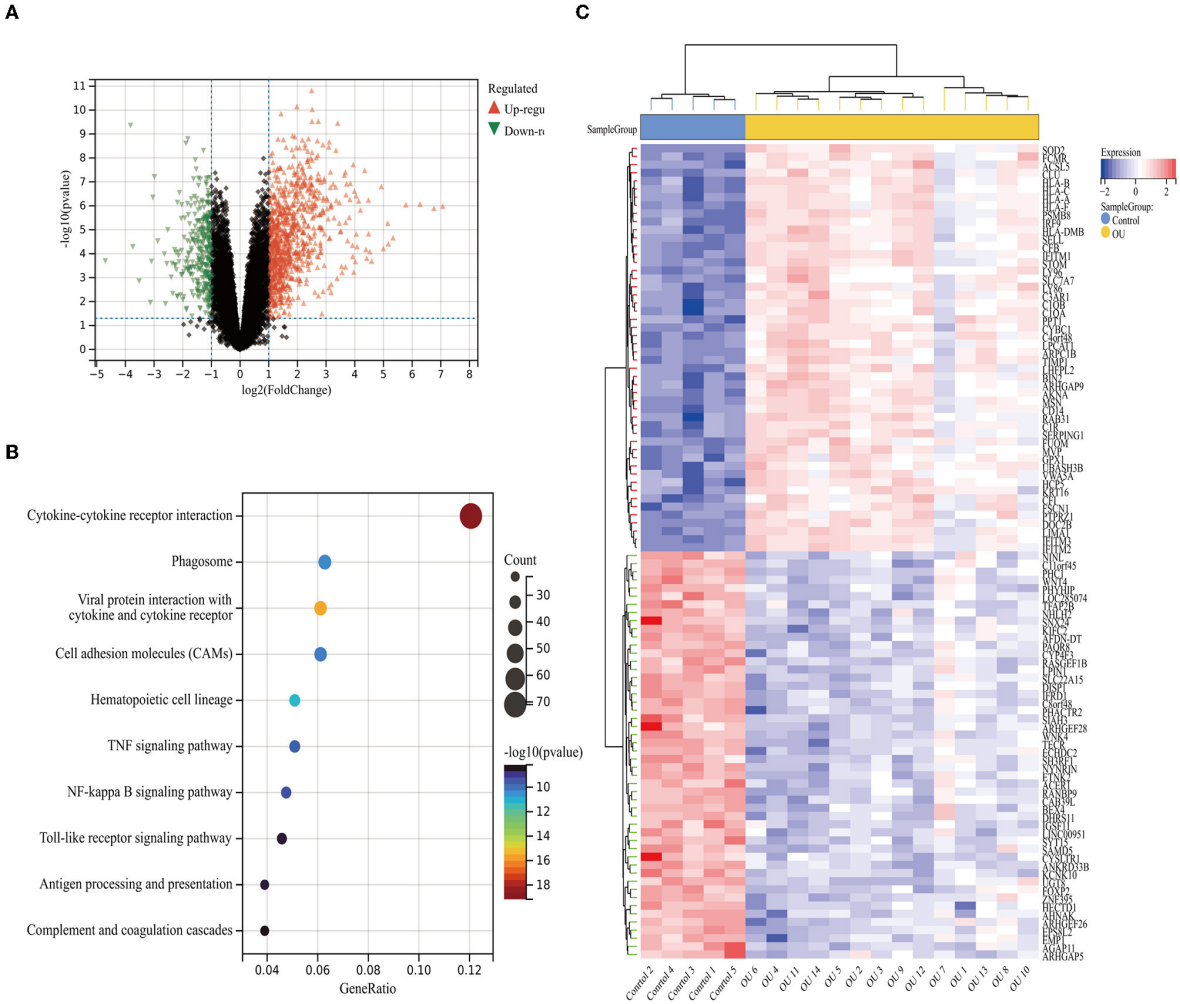
The DEGs between the high and low immune score groups. **(A)** Volcano plot of the DEGs. **(B)** Heat map of the DEGs. **(C)** Mostly enriched KEGG pathways. Identification of immune-related modules by WGCNA.

Using the limma package in R, we identified the DEGs between oral mucosal ulcers (high score) and normal oral mucosal tissues (low score). As shown in the volcano plots in Figure 2A, a total of 1,214 DEGs (up-regulated: 885, down-regulated: 329) were identified in the GSE37265 dataset. The top 100 DEGs (up-regulated: 50, down-regulated: 50) with the highest *P*-values are shown in cluster heat maps (Figure 2C). To investigate the related signaling pathways, we performed the KEGG pathway analysis. Significantly enriched KEGG pathways in the DEGs from GSE37265 are shown in Figure 2B. Among the pathways, the TNF signaling pathway, NF-κB signaling pathway, and toll-like receptor signaling pathway are well-known pathways related to the mechanism of wound inflammation. In addition, several other pathways have been suggested to be involved in inflammatory-related mechanisms in the immune system, such as cytokine-cytokine receptor interaction, phagosome regulation, and antigen processing and presentation. KEGG pathway analysis revealed that the most significantly enriched pathway was the cytokine-cytokine receptor interaction.

### Identification of Immune-Related Modules by WGCNA

**Figure 3:**
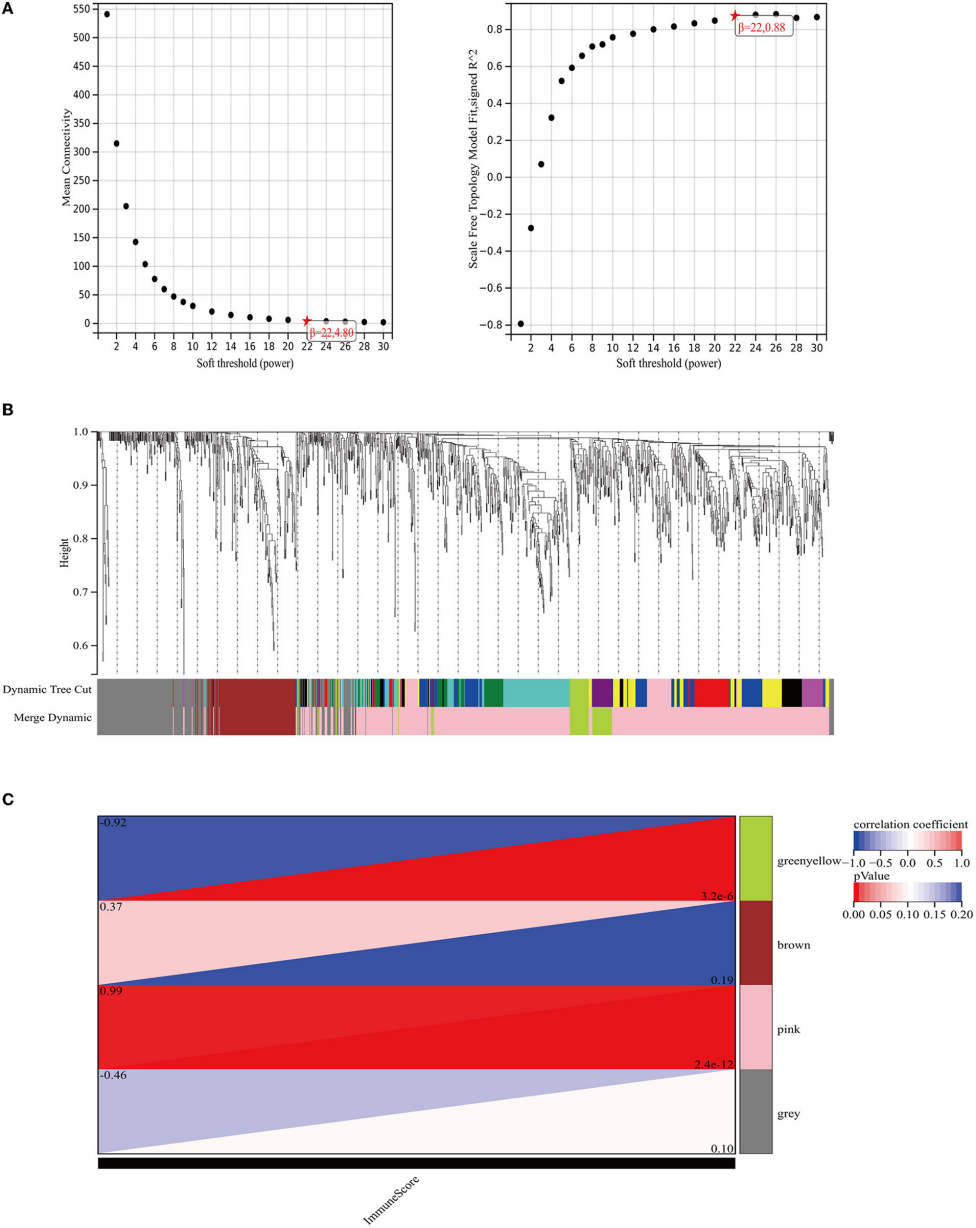
The weighted immune-related gene co-expression network. **(A)** The scale-free fit index for soft-thresholding powers. The right panel shows the relationship between the soft threshold and the scale-free R2. The left panel shows the relationship between the soft threshold and the mean connectivity. **(B)** A dendrogram of the DEGs clustered based on different metrics. Each branch in the figure represents one gene and each color below represents one co-expression module. **(C)** The heat map shows the correlation between the gene module and the immune score. The correlation coefficient in each cell, decreased in size from red to blue, represents the correlation between the gene module and the immune score.

WGCNA analysis was performed on 1214 DEGs (Figure 2). The soft-thresholding power in WGCNA was filtered to 22 (Figure 3A). As shown in Figure 3B, the first set of modules was obtained using the Dynamic Tree Cut algorithm; afterward, correlated modules were merged. Finally, four modules were identified on the basis of the average linkage hierarchical clustering and the soft-thresholding power. However, the gray module was used for housing the genes that were not co-expressed with other genes and thus cannot be assigned to any other module, which should be ignored in our study. The pink module and the green-yellow module were highly related to the immune score (Figure 3C). A significant positive correlation between the pink module and the immune score was observed, while a significant negative correlation existed between the green-yellow module and the immune score. The pink module contained 406 hub genes, while the green-yellow module contained 50 hub genes. Data in these two modules were selected for further analysis.

### Identification and Functional Annotations of DEGs in GSE80178

**Figure 4:**
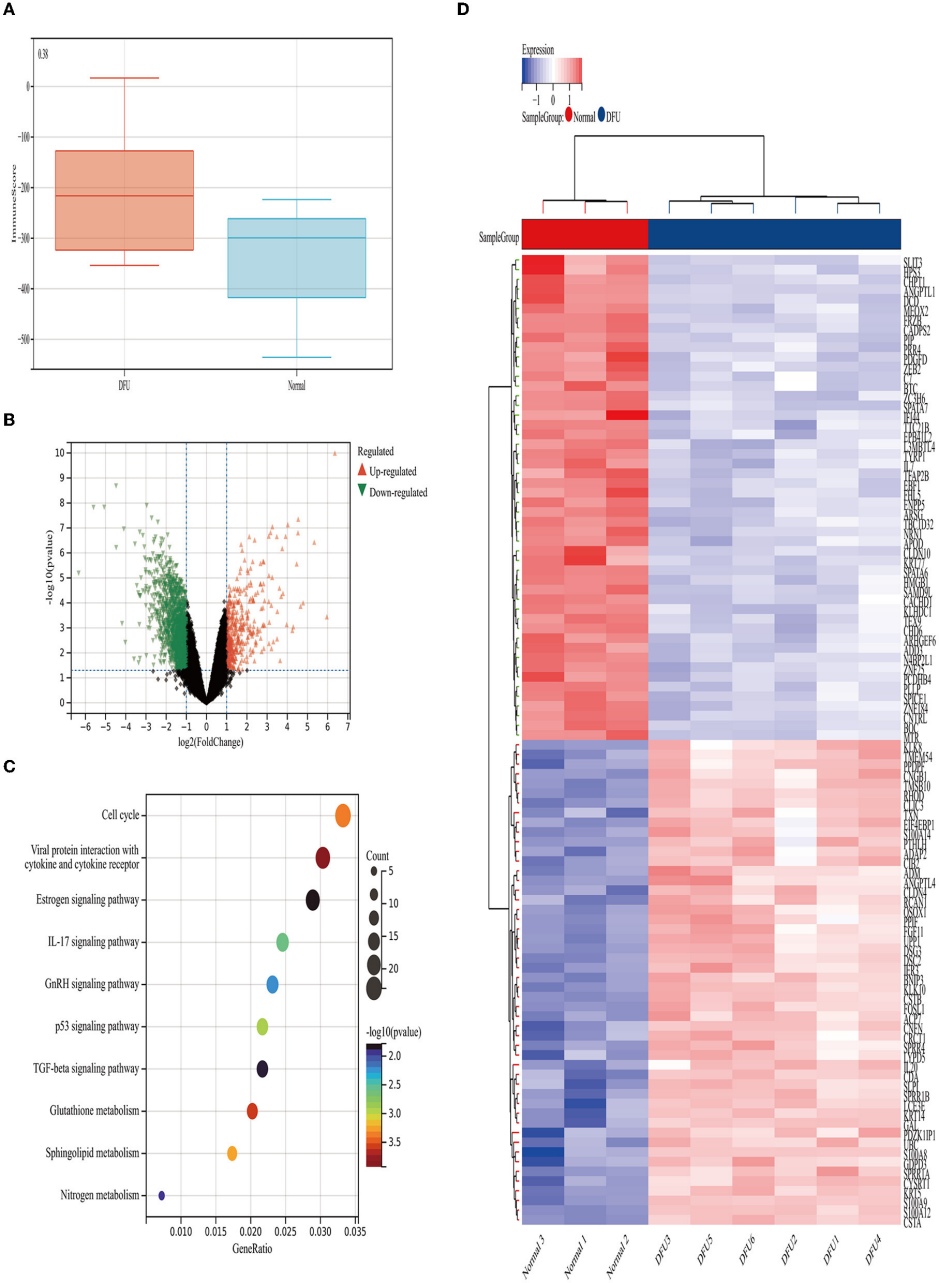
Immune scores and DEGs between the DFUs group and the normal foot skin group. **(A)** Distribution of the immune scores of DFUs and normal foot skin. **(B)** Volcano plot of the DEGs. **(C)** Heat map of the DEGs. **(D)** Mostly enriched KEGG pathways.

We obtained the GSE80178 dataset from the GEO database and found that the immune score had no significant difference between the DFUs and the control groups according to the ESTIMATE algorithm (*P* = 0.38) (Figure 4A). Distribution of immune scores were shown in Supplementary Table 3. Using the limma package in R, we identified the DEGs between the DFUs and the normal foot skin groups. As shown in the volcano plots, a total of 1825 DEGs (up-regulated: 409, down-regulated: 1416) were identified in the GSE80178 dataset (Figure 4B). The top 100 DEGs (up-regulated: 50, down-regulated: 50) with the highest *P*-values are shown in cluster heat maps (Figure 4D). KEGG pathway analysis is illustrated in Figure 4C. These DEG enrichment pathways were mainly concentrated in cellular processes and organismal systems, such as the cell cycle, interleukin-17 signaling pathway, and p53 signaling pathway. In addition, other pathways, such as glutathione metabolism, sphingolipid metabolism, and nitrogen metabolism, were primarily related to metabolic mechanism.

### Identification of Immune-Related Genes

**Figure 5:**
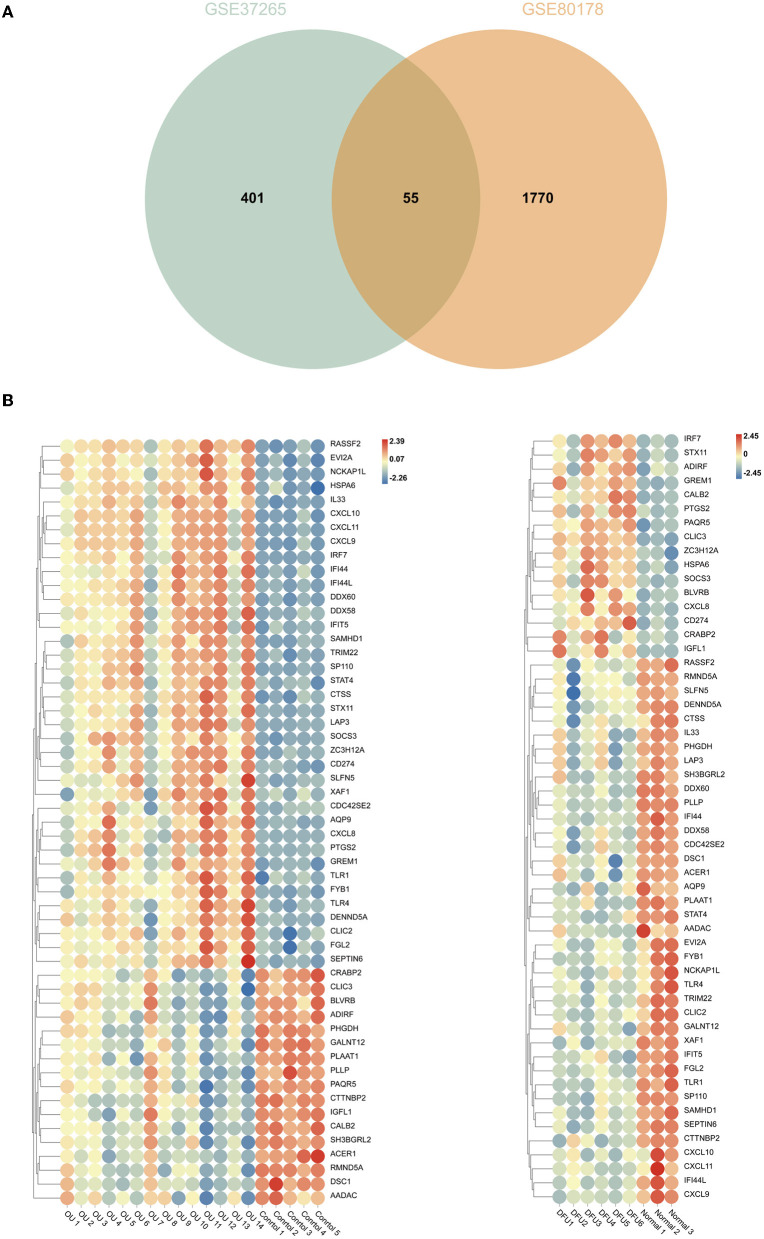
Comparison of gene expression profiles with immune-related genes. **(A)** Venn diagrams showing overlapping genes in GSE37265 and GSE80178 datasets. **(B)** Heat map of immune-related genes in GSE37265 and GSE80178 datasets.

The intersection of the analysis results of two data sets was obtained by Venn diagram (Figure 5A) and only 55 overlapping genes can be obtained in both groups. We defined these genes as immune-related genes and illustrated them in heat maps for each dataset. As shown in Figure 5B, almost all the genes were highly expressed in the oral ulcers group and lowly expressed in the DFUs group and vice versa. Overall, these immune-related genes showed opposite expression patterns in the two datasets.

### PPIs Among Immune-Related Genes

**Figure 6:**
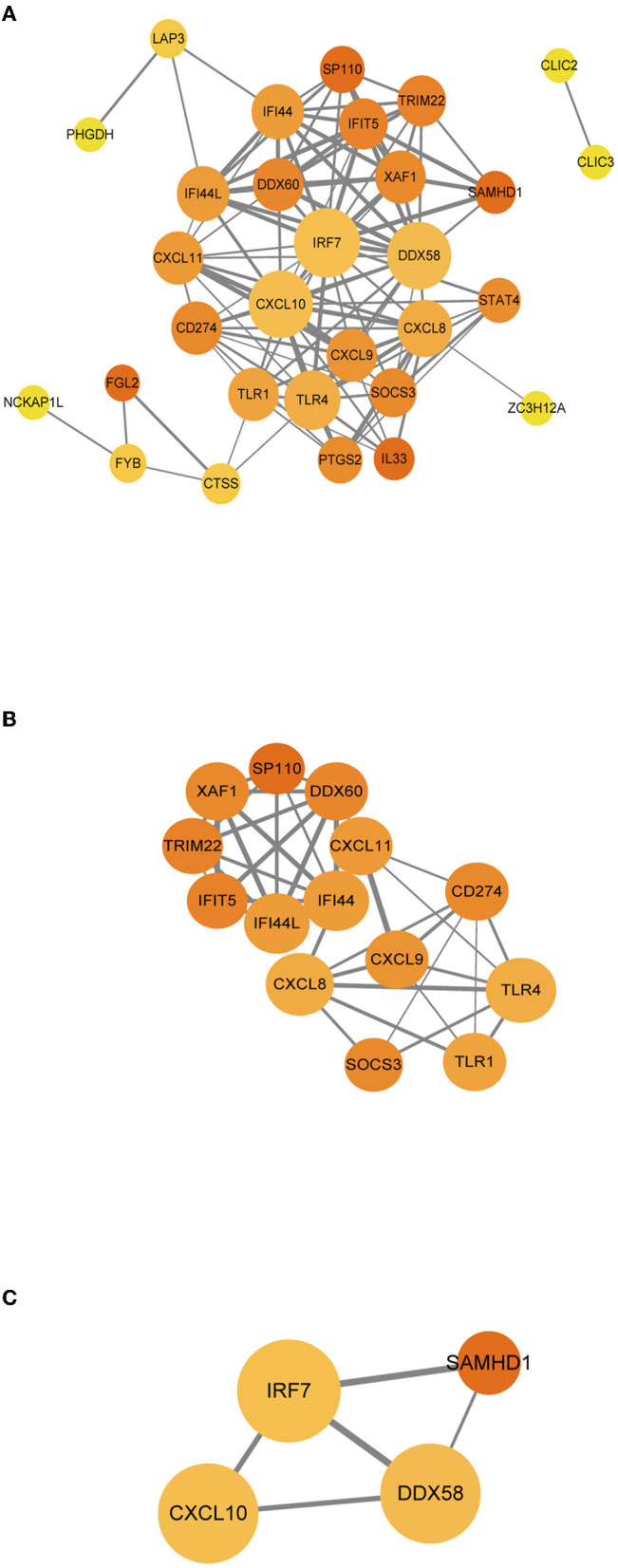
**(A–C)** PPI networks of immune-related genes. The node color in the PPI network reflects the clustering coefficient; the node size represents the number of interacting proteins with the designated protein, while the edge size indicates the combined score.

For better understanding of the interplay among the identified immune-related genes, we obtained the PPI network among the immune-related genes using the STRING tool. The network comprised 30 nodes and 116 edges (Figure 6A). Using MCODE, we selected the most significant modules for further analysis. In the first module (Figure 6B), 40 edges involving 14 nodes were formed in the network, where CXCL11, DDX60, IFI44, and IFI44L were remarkable nodes since they had the closest connections with the other members of the module. In the second module (Figure 6C), CXCL10, IRF7, and DDX58 together formed a closed-loop relationship and occupied central positions in the entire network.

### Validation of Hub Immune-Related Genes

**Figure 7:**
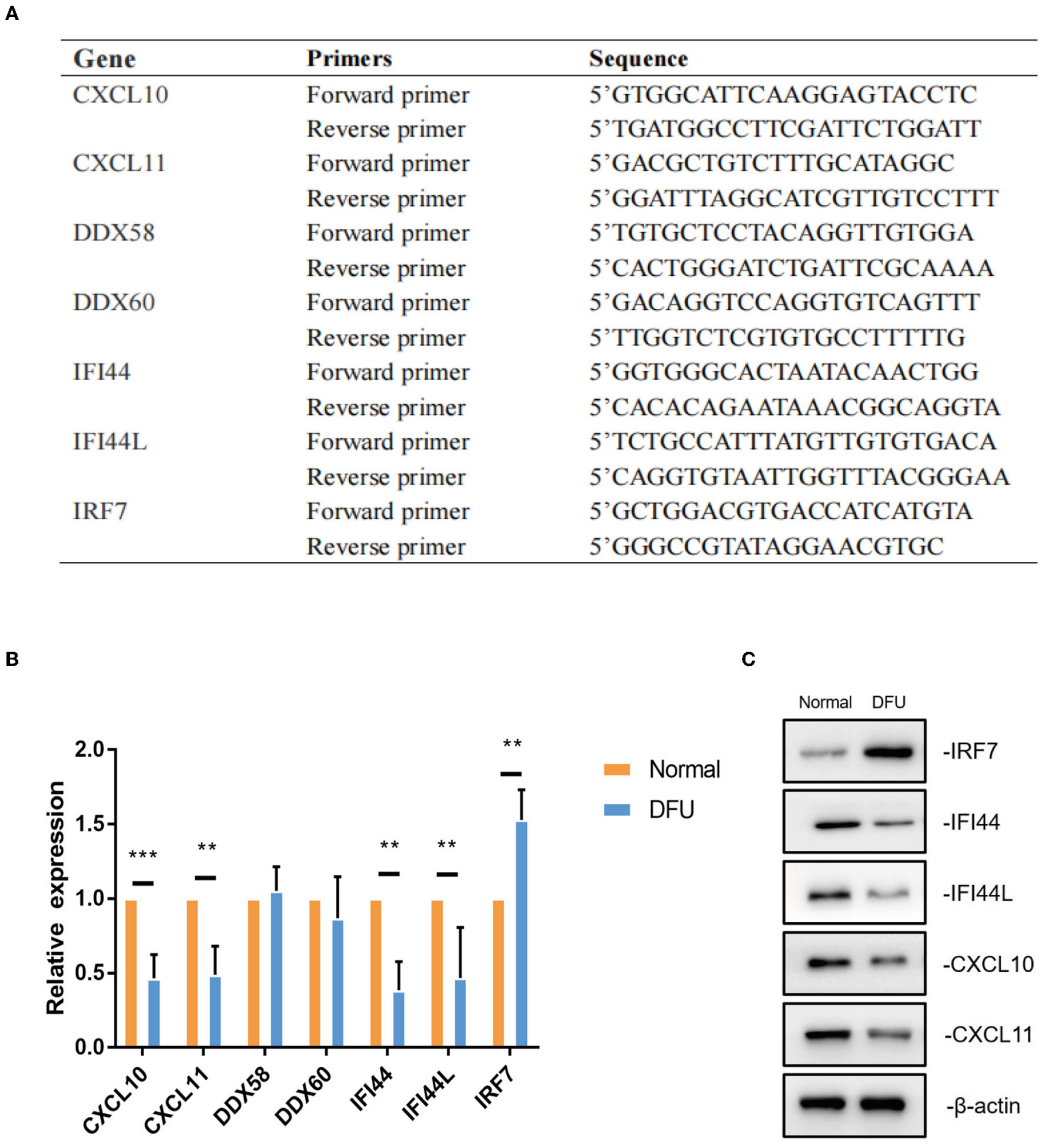
The validation of hub immune-related genes. **(A,B)** Gene expression of hub immune-related genes in DFU tissues were validated by real-time PCR. ** < 0.05,*** < 0.005. **(C)**CXCL11, IFI44, IFI44L, CXCL10 and IRF7 protein levels were measured by western blot (a representative blot, from three independent experiments).

To further test these hub immune-related genes,we performed a real-time PCR validation by comparing the normal wound tissues with the DFU tissues. In line with our bioinformatic results, CXCL11, IFI44, IFI44L, CXCL10 and IRF7 had a significant difference compared with normal wound tissues (*P* < 0.05) (Figure 7B). However, there was no statistical difference in DDX58 and DDX60 gene expression levels observed in the DFU group compared to the Normal group (*P* > 0.05). The results of western blot also indicated that CXCL11, IFI44, IFI44L, CXCL10, and IRF7 were indeed significantly different from normal tissues (Figure 7C).

## Discussion

DFUs and other chronic wounds are characterized by morphological and cellular changes; however, the underlying molecular biomarkers that result in these changes are poorly understood. Abnormal gene expression is closely related to a series of pathological conditions in DFUs. An altered mRNA expression is a valuable biomarker that plays an important role in the development of diabetes-related diseases (27, 28). Expanding our understanding of the mechanisms involved in diabetic wounds would improve the efficacy of wound healing and reduce the social burden. Unlike skin wounds, oral wounds heal more quickly and have fewer complications; however, our knowledge in this area is limited by the lack of detailed comparative analyses of humans. Considering that oral mucosa is recognized for quick and scarless healing, we decided to use oral ulcer as a model to explore the potential interventions related to wound immune activation during DFUs treatment.

In this study, we first analyzed the datasets of oral ulcers and DFUs using bioinformatics methods and found that the immune score of oral ulcers was significantly higher than that of the normal oral mucosa; however, there was no statistically significant difference between the immune scores of DFUs and that of normal foot skin. Afterward, KEGG function enrichment was performed on the differential genes in the two datasets, respectively, and it was found that the oral ulcers group was mainly enriched in inflammatory and immune-related pathways, while the DFU group was enriched in cellular metabolism-related pathways, lacking the activation of inflammatory and immune-related pathways. In conclusion, oral ulcers can significantly activate the immune system and initiate inflammatory responses; however, DFUs lack the corresponding activation.

Some studies have found that DFUs only have a low-intensity inflammation; in other words, the activation and function of the immune cells are reduced, which provides an inadequate inflammatory response and delay in the healing process (29). In our research, we found that M1 macrophages, M2 macrophages, and CD4 memory-activated T cells were the cell types with the highest degree of infiltration on the wound surface of oral ulcers, which were significantly up-regulated compared to the control group. Macrophages play crucial roles in the inflammatory stage of wound healing (11, 30). Macrophages undergo a phenotypic transition from M1 to M2, which ensures the inflammation resolution and the wound healing progression. This process was found to be out of control in diabetic ulcers, thus resulting in dysfunctional M1 macrophage accumulation and lack of M2 macrophage (31, 32). T cells are also critical for inflammation reduction and tissue remodeling. During inflammation, macrophages secrete chemokines, such as interferon-gamma, to attract T cells to the wound, which contributes to the formation of the initial pro-inflammatory microenvironment of the wound (33). However, activated natural killer (NK) cells and Tregs were the cell types with the lowest degree of wound infiltration, which were significantly down-regulated compared to the control group. Since NK cells inhibit wound healing, the depletion of NK cells in mice can improve the rate of wound healing (34). In contrast, wound healing was reported to be slower in Tregs-depleted mice than in wild-type mice (35). The down-regulation of Tregs in this study may be due to the transformation process of memory T cells. Tregs play an important role in maintaining skin homeostasis. Tregs secrete arginase and anti-inflammatory cytokines that promote the polarization of anti-inflammatory macrophages, inhibit inflammatory responses, and contribute to the matrix formation (36). Studies have demonstrated that NK cells are up-regulated, while Tregs are down-regulated in DFUs, and that different immune cell infiltrates would indeed cause different healing states (29).

Finally, we conducted the WGCNA analysis to find the immune-related modules and obtained 55 immune-related genes by the intersection with differential genes in the diabetes group. These genes were significantly differentiated genes in the two datasets; however, their overall expression patterns showed an opposite state. Through PPI analysis, 30 of them were closely related. By extracting the key modules, we finally identified 18 key immune-related genes. Among them, CXCL11, DDX60, IFI44, IFI44L, CXCL10, IRF7, and DDX58 were identified as the hub genes in this study. However, DDX58 and DDX60 showed no significant difference in the *in vitro* experiment, the rest genes were indeed found to be closely related to immune regulation after literature search. CXCL10 is an inflammatory chemokine secreted by monocytes, neutrophils, endothelial cells, keratinocyte, fibroblasts, mesenchymal cells, dendritic cells, and astrocytes (37). CXCL10, as one of the selective ligands of CXCR3, binds to CXCR3 and regulates immune responses by activating and recruiting leukocytes, such as T cells, eosinophils, monocytes, and NK cells (38). CXCL11 is also one of the selective ligands of CXCR3 and plays important roles in leukocyte homing and persistence of inflammation (39, 40). IFI44 was reported to negatively regulate innate immune and antiviral responses (41); it is also implicated in immune infiltration in the tumor microenvironment (42, 43). Previous studies have indicated a promising role of IFI44L in innate immunity, such as antiviral response, antitumor response, and inflammatory response (44–46). IRF7 is expressed in fibroblasts, B cells, lymphocytes, pDCs, and monocytes and plays important roles in regulating the innate and adaptive immune response (47). In this study, the above-mentioned genes showed a close relationship with immunity. Since most of the genes identified in this study have not been previously reported to be related with DFUs, there is a clear need to verify the functional importance and mechanistic roles of these genes in this pathological context. Based on the information obtained from literature, we are interested in further studies on the relationship between these genes and their functions as regulators of these debilitating chronic wounds.

## Conclusion

Overall, we have provided a comprehensive comparative analysis of molecular and cellular mechanisms by studying different wound healing processes in the oral cavity and skin, ultimately highlighting the fundamental mechanisms of inflammation and repair in human being, thereby providing insights for treating chronic and unhealed wounds.

## Data Availability Statement

The original contributions presented in the study are included in the article/Supplementary Material, further inquiries can be directed to the corresponding authors.

## Ethics Statement

The studies involving human participants were reviewed and approved by the Institutional Review Board of The First Affiliated Hospital of Sun Yat-sen University. The patients/participants provided their written informed consent to participate in this study.

## Author Contributions

ZH and JZ designed the research. YR, HY, HX, SL, PW, ZW, YZ, WZ, BT, JZ, and ZH performed the experiment. YR, HY, and ZH wrote the manuscript. YR, HY, and HX researched data. BT, JZ, and ZH reviewed/edited the manuscript. All authors contributed to the article and approved the submitted version.

## Funding

This work was supported by research grants 82172213 (ZH), 82072180 (JZ), 82072181 and 81871565 (BT) from National Natural Science Foundation of China, research grant 2019A1515012208 (ZH) from Guangdong Provincial Natural Science Foundation of China, and research grants 2013001 and 2018002 (JZ) from the Sun Yat-sen University Clinical Research 5010 Program, and Kelin New Star Talent Program of The First Affiliated Hospital of Sun Yat-sen University (ZH).

## Conflict of Interest

The authors declare that the research was conducted in the absence of any commercial or financial relationships that could be construed as a potential conflict of interest.

## Publisher's Note

All claims expressed in this article are solely those of the authors and do not necessarily represent those of their affiliated organizations, or those of the publisher, the editors and the reviewers. Any product that may be evaluated in this article, or claim that may be made by its manufacturer, is not guaranteed or endorsed by the publisher.
