# Supplementary material for: Bioinformatic Analysis Reveals Hub Immune-Related Genes of Diabetic Foot Ulcers
Source: Front Surg. 2022 Apr 5;9:878965. doi: 10.3389/fsurg.2022.878965 (PMC9016148; doi:10.3389/fsurg.2022.878965)
Supplement: Supplementary file 1 [file Table_1.DOCX]

| **Table S1. Antibodies for Western blot** | | |
| --- | --- | --- |
| **Antibody** | **Source** | **Catalog No.** |
| IRF7 | Invitrogen | 700229 |
| CXCL10 | Cell signalling | 14969S |
| CXCL11 | abcam | ab181035 |
| IFI44 | abcam | ab172499 |
| IFI44L | abcam | ab161220 |
| Actin Beta | Proteintech | 60008-1-Ig |
| \| HRP- anti-mouse IgG \| Jackson \| 115-035-003 \| \| --- \| --- \| --- \| | Jackson | 115-035-003 |
| HRP- anti-rabbit IgG | Jackson | 111-035-003 |
