# Supplementary material for: Bioinformatic Analysis Reveals Hub Immune-Related Genes of Diabetic Foot Ulcers
Source: Front Surg. 2022 Apr 5;9:878965. doi: 10.3389/fsurg.2022.878965 (PMC9016148; doi:10.3389/fsurg.2022.878965)
Supplement: Supplementary file 2 [file Table_2.DOCX]

| ID | StromalScore | ImmuneScore | ESTIMATEScore |
| --- | --- | --- | --- |
| OU1 | 1097.109003 | 1780.983918 | 2878.092922 |
| OU2 | 137.9464784 | 2095.853997 | 2233.800475 |
| OU3 | 821.1191317 | 2072.460305 | 2893.579436 |
| OU4 | 870.3924349 | 2592.407433 | 3462.799868 |
| OU5 | 1187.262856 | 2267.718606 | 3454.981462 |
| OU6 | 483.2247655 | 2424.064968 | 2907.289733 |
| OU7 | -450.6300375 | 1043.495497 | 592.8654599 |
| OU8 | 753.1129701 | 1935.914884 | 2689.027854 |
| OU9 | 331.7031735 | 2429.165603 | 2760.868776 |
| OU10 | 569.238843 | 2474.737951 | 3043.976794 |
| OU11 | 1146.32526 | 3191.275386 | 4337.600646 |
| OU12 | 361.0284133 | 2867.981324 | 3229.009737 |
| OU13 | 1150.845647 | 1938.647154 | 3089.492801 |
| OU14 | 1606.039229 | 3107.064875 | 4713.104104 |
| Normal-1 | -509.8925613 | 243.636713 | -266.2558483 |
| Normal-2 | -762.2029411 | 459.380025 | -302.8229161 |
| Normal-3 | -1211.074715 | 41.25331017 | -1169.821405 |
| Normal-4 | -651.8570976 | 787.7245839 | 135.8674862 |
| Normal-5 | -781.8384931 | 11.06902095 | -770.7694721 |
