# Supplementary material for: Bioinformatic Analysis Reveals Hub Immune-Related Genes of Diabetic Foot Ulcers
Source: Front Surg. 2022 Apr 5;9:878965. doi: 10.3389/fsurg.2022.878965 (PMC9016148; doi:10.3389/fsurg.2022.878965)
Supplement: Supplementary file 3 [file Table_3.DOCX]

| ID | StromalScore | ImmuneScore | ESTIMATEScore |
| --- | --- | --- | --- |
| DFU1 | -442.0392922 | -353.8523224 | -795.8916146 |
| DFU2 | -204.1021009 | 16.76097988 | -187.3411211 |
| DFU3 | -608.752686 | -108.9722023 | -717.7248882 |
| DFU4 | -472.5543737 | -348.27809 | -820.8324637 |
| DFU5 | -428.5765638 | -182.7307313 | -611.3072951 |
| DFU6 | -400.2978642 | -249.7765844 | -650.0744486 |
| Normal-1 | -17.10864009 | -535.5202927 | -552.6289328 |
| Normal-2 | 170.8955755 | -299.3867629 | -128.4911875 |
| Normal-3 | 276.6723138 | -223.519359 | 53.15295483 |
